# Reply to ‘Management of noncompaction requires optimisation’

**DOI:** 10.1530/ERP-19-0037

**Published:** 2019-05-31

**Authors:** Sothinathan Gurunathan, Roxy Senior

**Affiliations:** 1 Department of Cardiology, Northwick Park Hospital, Harrow, UK; 2 Department of Cardiology, Royal Brompton Hospital, National Heart and Lung Institute, Imperial College, London, UK

**Dear Editor,**

We thank Josef Finsterer and Claudia Stöllberger for their comments, published as a Letter in this issue of *Echo Research and Practice* (volume 6, page L1, https://doi.org/10.1530/ERP-19-0008), which we read with great interest (1).

Standard echocardiography is the initial diagnostic modality of choice for left ventricular non-compaction (LVNC) in both index patients and family members. Two-dimensional echocardiography may show both trabeculae and deep intertrabecular recesses in the LV myocardium. Unconventional views may be needed to image the more apical segments of the myocardium. In obese patients or patients with lung disease who may have poor acoustic windows, contrast-enhanced echocardiography enhances the endocardial border definition, resulting in improved visualization of the non-compacted apex and mid-walls. In instances where the apex is foreshortened, 3D TTE allows clear visualization of all myocardial segments including the apex. We believe that echocardiography is sufficient for the diagnosis of LVNC in experienced hands in most cases, particularly with 3D evaluation and contrast enhancement when indicated.

We agree that the most common presentation for LVNC is as an incidental finding on cardiac imaging. We use the word ‘classical’ as referring to most known about presentation, not the most common presentation.

Regarding the possibility of atrial fibrillation, there was no history of palpitations in our patient. We have stated that his electrocardiogram and 48-h tape were both normal. The atria were also of normal size (2).

Following presentation with a stroke, it has been mentioned in the text that our patient had occlusion of the M1 and M2 branches of the middle cerebral artery on computed tomography (CT) intracranial angiography. The patient underwent urgent thrombectomy for this. We also mentioned that our patient underwent decompressive craniotomy; following clinical deterioration, a CT was conducted 2 days later, which demonstrated haemorrhagic transformation within the infarct and associated mass effect (2).

Our patient was anticoagulated 6 months after the stroke, with warfarin. There are no clear guidelines for anticoagulation in patients with LVNC. Some investigators recommend long-term prophylactic anticoagulation for all patients with LVNC. However, some recommend anticoagulation where there is left ventricular dysfunction, atrial fibrillation, a history of thromboembolic complications, known ventricular thrombi, chamber dilatation or evidence of spontaneous contrast (3, 4).

Undoubtedly, in retrospect, it would have been better to have commenced this patient on anticoagulation. However, we have published this case report to stimulate discussion on the topic of anticoagulation in LVNC.

## Declaration of interest

Roxy Senior is an associate editor for *Echo Research and Practice*. He was not involved in the review or editorial process for this paper, on which he is listed as an author. S Gurunathan declares that there is no conflict of interest that could be perceived as prejudicing the impartiality of this reply.

## Funding

This work did not receive any specific grant from any funding agency in the public, commercial or not-for-profit sector.
